# Small Molecule Targeted Therapy for Hepatocellular Carcinoma: From Multikinase Inhibition to Cancer Stem Cell Eradication Strategies

**DOI:** 10.32604/or.2026.087514

**Published:** 2026-10-28

**Authors:** Jian Wang, Chunxiu Suo, Yu Xie, Renjun Gu, Nan Lu, Xinyan Zhang, Xiaojuan Liu

**Affiliations:** 1 National Administration of Traditional Chinese Medicine Institutional Service Center, Beijing, China; 2 Department of Critical Care Medicine, Rongcheng Shidao People’s Hospital (Formerly Rongcheng No.2 People's Hospital), Rongcheng, China; 3 PLA Rocket Force Characteristic Medical Center, Beijing, China; 4 School of Chinese Medicine, Nanjing University of Chinese Medicine, Nanjing, China; 5 State Key Laboratory of Reproductive Medicine and Offspring Health, Clinical Center of Reproductive Medicine, The First Affiliated Hospital of Nanjing Medical University, Nanjing, China; 6 Department of Hepatology and Hematology, Shaanxi Provincial Hospital of Traditional Chinese Medicine, Xi’an, China

**Keywords:** Hepatocellular carcinoma, cancer stem cells, multikinase inhibitors, drug resistance, small molecule inhibitors, wnt β-catenin pathway, combination therapy

## Abstract

Hepatocellular carcinoma (HCC) remains a leading cause of cancer-related death worldwide, posing a significant global health burden. Multikinase inhibitors (MKIs) such as sorafenib and lenvatinib are standard therapies for advanced HCC, yet most patients eventually develop drug resistance and tumor relapse, largely attributed to the presence of cancer stem cells (CSCs). These cells, which possess self-renewal capacity and intrinsic resistance to conventional treatments, drive tumor heterogeneity and recurrence. This review examines the molecular features of HCC CSCs and their regulatory networks, including Wnt/β-catenin, Phosphoinositide 3-kinase (PI3K)/Akt/Mammalian target of rapamycin (mTOR), and Notch signaling pathways. CSCs drive tyrosine kinase inhibitor (TKI) resistance through mechanisms such as Caveolin 1 (CAV1)-mediated dormancy and the CD133/Myosin heavy chain 9 (MYH9)/β-catenin axis, which sustain survival and proliferation despite targeted therapy. Recent preclinical studies have identified small molecule inhibitors targeting CSC-specific pathways and markers, including C504244, 4-MU, bestatin, OSU-CG5, and CL26, which show promise in eradicating CSC populations. Combination strategies that pair TKIs with these agents demonstrate synergistic effects, particularly when delivered via nanoparticle systems, which enhance drug bioavailability and tumor targeting. However, major barriers to clinical translation include CSC heterogeneity, microenvironmental adaptation, and the lack of reliable biomarkers for patient stratification. The shift from multikinase inhibition to CSC eradication represents a promising paradigm for transforming HCC into a more manageable disease, potentially improving long-term outcomes and reducing relapse rates.

## 1 Introduction

### 1.1 Epidemiological Landscape and Clinical Challenges

Hepatocellular carcinoma (HCC) accounts for approximately 75–85% of all primary liver cancers. It is currently the third leading cause of cancer-related death worldwide. The global burden of this disease continues to rise, driven largely by the increasing prevalence of metabolic dysfunction-associated steatotic liver disease (MASLD). Concurrently, chronic infections with hepatitis B virus and hepatitis C virus remain major risk factors, especially in East Asia and sub-Saharan Africa. In Western countries, the rapid increase in MASLD-related HCC reflects the ongoing obesity epidemic [1].

The clinical management of HCC is highly challenging for two main reasons. First, HCC is a highly heterogeneous tumor, meaning that different parts of the same tumor or tumors from different patients can exhibit distinct morphological and behavioral characteristics. Second, most patients already have underlying chronic liver disease, which limits their tolerance to many treatments [2]. For patients diagnosed at an early stage, which is BCLC stage 0 or A, curative interventions are possible. These include surgical resection, liver transplantation, and local ablation. In these patients, the five-year survival rate can reach 60 to 80 percent [3].

Unfortunately, the outlook is far bleaker for the majority of patients. More than 70 percent of HCC patients are diagnosed at an intermediate or advanced stage, corresponding to BCLC stage B, C, or D, at which point curative options are no longer feasible [4]. Systemic therapy becomes the mainstay of treatment. Even with the best available therapies, the median overall survival (OS) for advanced HCC is only about 12 to 18 months [5]. Once extrahepatic metastasis or major vascular invasion occurs, the five-year survival rate drops to a mere 3 to 5 percent. Moreover, the recurrence rate after resection or ablation is extremely high, ranging from 70 to 80 percent within five years. These statistics highlight an urgent need for more effective therapeutic strategies that can achieve long-lasting tumor control [6].

To ensure a systematic and comprehensive coverage of the relevant literature, we conducted a structured search of PubMed for articles published between January 2000 and June 2026. The search terms included combinations of keywords: “hepatocellular carcinoma”, “cancer stem cells”, “multikinase inhibitor”, “drug resistance”, “small molecule inhibitor”, “Wnt/β-catenin”, “nanomedicine”, and “combination therapy”. We prioritized original research articles, clinical trials, and high-quality review articles published in peer-reviewed journals. Articles were included if they provided mechanistic insights into CSC regulation, TKI resistance mechanisms, or preclinical evaluation of CSC-targeted small molecules. Conference abstracts, preprints, and non-English articles were excluded from the analysis.

### 1.2 Limitations of Current Systemic Therapies

Over the past two decades, systemic therapy for advanced HCC has evolved significantly. We have transitioned from an era with no effective drugs to one with several approved multikinase inhibitors and immune checkpoint inhibitors (ICIs). However, each of these drug classes has intrinsic limitations that collectively create a therapeutic ceiling [7].

Multikinase inhibitors were the first targeted agents to demonstrate benefit in HCC. Sorafenib was approved in 2007 and established the foundation of molecular therapy for this disease. Later, lenvatinib was shown to be non-inferior to sorafenib with a higher objective response rate. Regorafenib, cabozantinib, and ramucirumab were subsequently approved as second-line options [8]. Despite these advances, the objective response rate of MKIs as single agents rarely exceeds 30 percent by standard response criteria [9]. Importantly, most patients develop acquired resistance within six months of starting treatment. While the mechanisms of resistance are complex and involve alternative pathway activation (e.g., Mesenchymal epithelial transition factor [MET], Epidermal growth factor receptor [EGFR]) and enhanced drug efflux, a key emerging concept is the role of therapy-resistant cancer stem cells (CSCs) in driving treatment failure. We discuss this mechanism in detail in Section 3, but briefly, CSCs reside at the top of the tumor hierarchy and are intrinsically resistant to Tyrosine kinase inhibitors (TKIs), providing a reservoir for tumor regrowth after initial response [10].

ICIs represent another major breakthrough. The combination of atezolizumab, an anti-programmed death ligand 1 (PD-L1) antibody, plus bevacizumab, an anti-Vascular endothelial growth factor (VEGF) antibody, has become a global standard of care for first-line treatment [11]. However, ICIs face two major challenges. First, primary resistance is common. About 60–70% of HCC patients do not achieve objective responses. This is largely attributable to an immunosuppressive tumor microenvironment (TME) that contains abundant regulatory T cells (Tregs), tumor-associated macrophages (TAMs) with an M2 polarization state, and exhausted CD8-positive T cells. Second, and more concerning, emerging evidence shows that patients with MASLD-related HCC derive substantially less benefit from ICIs compared to those with viral hepatitis related HCC [12]. This appears to be driven by aberrant T cell activation, where MASLD-associated CD8-positive Programmed cell death protein-1 (PD-1)-positive T cells paradoxically promote tumor progression rather than suppress it. This etiology-dependent heterogeneity complicates treatment selection and underscores the need for personalized approaches [13].

A common limitation of both multikinase inhibitors and ICIs is that neither directly targets the tumor-initiating cell population, namely the CSCs. These therapies can shrink the bulk tumor mass, but they leave behind a resilient reservoir of CSCs that survive the therapeutic insult [14]. These residual CSCs then repopulate the tumor, leading to recurrence, often accompanied by acquired resistance to prior therapies. This fundamental limitation leads to an inescapable conclusion: eliminating proliferating tumor cells alone is not sufficient. The therapeutic paradigm must evolve to include CSC eradication [15].

### 1.3 The Emergence of the CSC Theory in HCC

The CSC theory proposes that tumors are organized in a hierarchical manner. A small subset of cells within the tumor, known as CSCs, possesses unique capacities for self-renewal, multipotent differentiation, and sustained tumorigenicity. This theory was first validated in hematologic malignancies and later extended to solid tumors including HCC [16]. The CSC model provides a compelling explanation for several long-standing clinical puzzles: why tumors recur after seemingly complete remission [17], why metastasis can occur even from small primary lesions, and why conventional therapies eventually fail [18].

Over the past decade, researchers have identified multiple markers to isolate and characterize CSCs in HCC. These include cell surface markers such as CD133, CD90, Epithelial cell adhesion molecule (EpCAM), CD44, CD13, CD47, and Leucine-rich repeat containing G protein coupled receptor 5 (LGR5). Intracellular markers such as aldehyde dehydrogenase (ALDH) activity and CK19 have also been used [19]. In addition, transcription factors including SRY box transcription factor 2 (SOX2), Octamer binding transcription factor 4 (OCT4), Homeobox protein NANOG (NANOG), Spalt like transcription factor 4 (SALL4), and B cell specific Moloney murine leukemia virus integration site 1 (BMI 1) are highly expressed in CSCs and help maintain the stem cell state [20]. Importantly, HCC CSCs are not a homogeneous population. They exhibit phenotypic and functional heterogeneity. For example, EpCAM-positive CSCs tend to display an epithelial phenotype and are more proliferative whereas CD90-positive CSCs exhibit a mesenchymal like profile with enhanced migratory and invasive capabilities. This subpopulation diversity suggests that multiple CSC subsets may coexist within a single tumor [21], each contributing to different aspects of tumor progression and therapeutic resistance [22].

The CSC phenotype is orchestrated by a set of developmentally conserved signaling pathways. These include the Wnt/β-catenin pathway, the Phosphoinositide 3 kinase (PI3K)/Akt/Mammalian target of rapamycin (mTOR) pathway, the Nuclear factor kappa B (NF-κB) pathway, the Notch pathway, the Hedgehog pathway, the Janus kinase (JAK)/signal transducer and activator of transcription (STAT) pathway, and the Transforming growth factor beta (TGF beta) SMAD pathway. Among these, the Wnt β-catenin pathway occupies a central position. Hyperactivation of this pathway maintains the undifferentiated state, promotes self-renewal, and drives chemotherapy resistance. Importantly, the activation of these pathways is not entirely cell-intrinsic. Cues from the TME such as hypoxia, cytokines from TAMs, and exosomes secreted by cancer-associated fibroblasts (CAFs) further reinforce CSC stemness. This close interaction between CSCs and their niche makes the tumor ecosystem highly adaptive and resilient to therapy [23].

The recognition of CSCs as the roots of HCC has catalyzed a paradigm shift in therapeutic thinking. The traditional approach, which focuses on killing rapidly dividing tumor cells, can be described as a cytoreductive paradigm [24]. The emerging approach focuses on eliminating the CSC population. The underlying logic is straightforward yet transformative: if CSCs are the source of tumor initiation, maintenance, and recurrence, then eliminating this population should achieve more durable responses and potentially curative outcomes (Fig. 1) [25]. Accordingly, recent research efforts have increasingly focused on developing small molecule inhibitors that target CSC-specific markers, signaling pathways, metabolic vulnerabilities, and microenvironmental interactions. These agents, when used alone or in combination with conventional multikinase inhibitors or ICIs, hold promise for dismantling the CSC reservoir and overcoming therapeutic resistance [26].

**Figure 1:**
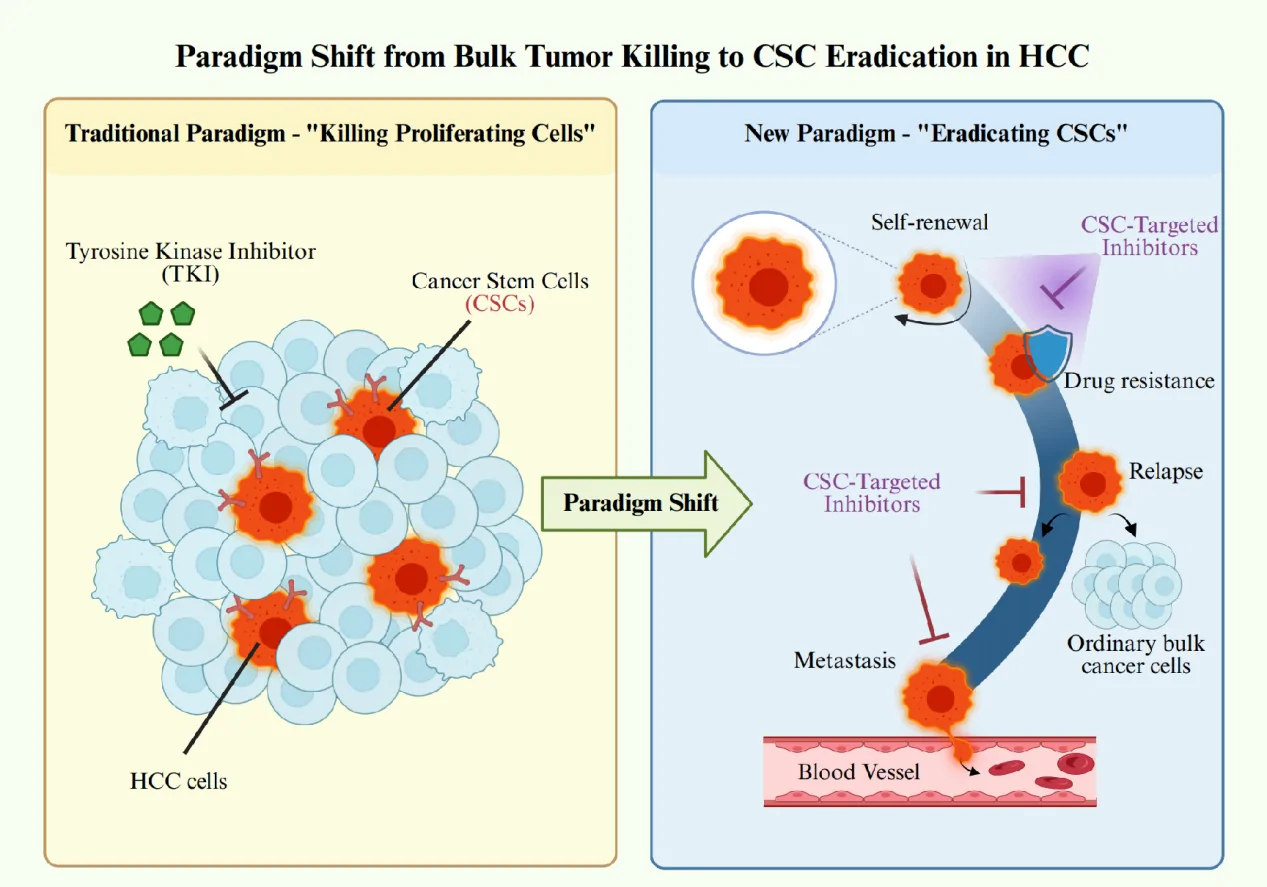
Paradigm shift from bulk tumor killing to cancer stem cells (CSC) eradication in hepatocellular carcinoma (HCC). This diagram shows the strategic shift in HCC therapy from targeting proliferating tumor cells to eradicating CSCs. The left side depicts how conventional therapies shrink the tumor mass but leave resistant CSCs, leading to relapse. The right side shows that new CSC-targeted agents aim to eliminate the root cause of recurrence for more durable outcomes. This figure was created using BioRender.

## 2 Molecular Features and Regulatory Networks of CSCs in HCC

### 2.1 Identification Markers of HCC CSCs

The identification and isolation of CSCs in HCC rely on a set of well-established markers. These markers can be broadly categorized according to their cellular localization and function into cell surface markers, intracellular markers, and transcription factors. Each category offers a distinct perspective for characterizing the CSC population.

Cell surface markers are the most widely used tools for isolating CSCs from tumor tissues or cell lines. In HCC, several such markers have been validated. CD133, one of the first markers identified, remains among the most extensively studied. CD133-positive cells isolated from HCC tumors exhibit enhanced self-renewal capacity, heightened tumorigenicity in mouse models, and increased resistance to conventional chemotherapy and targeted therapy. CD90, another important cell surface marker, is associated with a more invasive and metastatic phenotype. EpCAM is of particular interest as it marks CSCs with an epithelial phenotype. These EpCAM positive cells tend to be more proliferative and are enriched in the tumor bulk. CD44, a well-known marker in multiple cancer types, identifies CSCs in HCC. CD44-positive cells exhibit enhanced migratory capacity and are frequently associated with poor clinical outcomes. CD13 serves as a marker for quiescent CSCs. These slow-cycling cells evade conventional therapies that target rapidly dividing cells. CD47 functions as a “do not eat me” signal, enabling CSCs to evade phagocytosis by macrophages. LGR5, a target gene of the Wnt signaling pathway, marks a subset of CSCs with high self-renewal activity. Collectively, these cell surface markers not only facilitate the isolation and study of CSCs but also serve as potential therapeutic targets [27]. However, it is important to recognize that these markers are not static or universally expressed across all HCC patients. The expression of CSC markers is dynamically regulated by the tumor microenvironment (TME), therapeutic pressure, and the evolutionary selection of resistant clones. For instance, CD133 expression is upregulated in response to sorafenib treatment, representing an adaptive survival mechanism rather than a pre-existing property [28,29]. This dynamic plasticity implies that reliance on a single marker for CSC identification or therapeutic targeting may be insufficient; a multi-marker panel or a functional approach (e.g., ALDH activity) may be more robust across different clinical contexts [30]. Furthermore, the heterogeneity of marker expression across different HCC etiologies (e.g., MASLD vs. viral hepatitis) adds another layer of complexity that must be considered when designing CSC-targeted therapies.

Intracellular markers provide additional tools for CSC identification. ALDH, an enzyme involved in detoxification and drug metabolism, serves as a functional marker of CSCs across many tumor types, including HCC [31]. ALDH-positive cells exhibit enhanced resistance to chemotherapy, as the enzyme metabolizes various chemotherapeutic agents. demonstrate greater tumor-initiating capacity in animal models. CK19, another intracellular marker, is associated with an epithelial differentiation state and is frequently combined other markers to define specific CSC subpopulations [32].

Transcription factors play a central role in maintaining the stem cell state. These proteins bind to DNA and regulate the expression of genes involved in self-renewal and pluripotency. SOX2, OCT4, and NANOG form a core transcription factor network that maintains embryonic stem cell characteristics. In HCC CSCs, these factors are aberrantly expressed, driving tumor initiation and progression. SALL4 is another transcription factor that is frequently overexpressed in HCC and correlates with poor prognosis [33]. It promotes CSC self-renewal and drug resistance. BMI-1, a component of the polycomb repressive complex, is essential for maintaining the stem cell pool. It functions by repressing tumor suppressor genes and cell cycle regulators. (Table 1).

**Table 1:** Classification of HCC CSC markers.

Category	Markers	Functional Significance
Cell surface markers	CD133	Maintains CSC self-renewal, closely associated with drug resistance and recurrence
Cell surface markers	CD90	Marks mesenchymal phenotype CSCs, enhances migration and metastatic capacity
Cell surface markers	EpCAM	Marks epithelial phenotype CSCs, promotes proliferation and tumor initiation
Cell surface markers	CD44	Involved in cell adhesion and migration, mediates chemotherapy resistance
Cell surface markers	CD13	Marks quiescent CSCs, enables escape from conventional cytotoxic drugs
Cell surface markers	CD47	Inhibits macrophage phagocytosis, mediates immune evasion
Cell surface markers	LGR5	Wnt pathway target gene, maintains CSC self-renewal
Cell surface markers	CD24	Promotes CSC proliferation, associated with poor prognosis
Cell surface markers	CD166	Involved in cell cell adhesion, enhances CSC tumorigenicity
Intracellular markers	ALDH	Metabolic detoxification, clears chemotherapeutic agents, maintains CSC activity
Intracellular markers	CK19	Marks epithelial differentiation state, indicates invasive phenotype
Intracellular markers	GPC3	Regulates Wnt signaling, promotes CSC proliferation and survival
Transcription factors	SOX2	Maintains pluripotency and self-renewal, inhibits differentiation
Transcription factors	OCT4	Regulates stemness related gene expression, maintains undifferentiated state
Transcription factors	NANOG	Promotes CSC self-renewal, resists apoptosis
Transcription factors	SALL4	Maintains embryonic stem cell properties, drives tumor progression
Transcription factors	BMI-1	Inhibits cellular senescence and apoptosis, maintains CSC pool
Transcription factors	KLF4	Regulates CSC stemness, involved in epithelial mesenchymal transition
Transcription factors	MYC	Promotes CSC proliferation, regulates metabolic reprogramming

Abb: CD→Cluster of Differentiation, EpCAM→Epithelial cell adhesion molecule, LGR5→Leucine rich repeat containing G protein coupled receptor 5, ALDH→Aldehyde dehydrogenase, GPC3→Glypican 3, SOX2→SRY box transcription factor 2, OCT4→Octamer binding transcription factor 4, NANOG→Homeobox protein NANOG, SALL4→Spalt like transcription factor 4, BMI-1→B cell specific Moloney murine leukemia virus integration site 1, KLF4→Kruppel like factor 4, MYC→Myelocytomatosis oncogene.

### 2.2 Signaling Pathways That Regulate CSC Stemness

The stemness properties of HCC CSCs are orchestrated by a set of evolutionarily conserved signaling pathways. These pathways, normally active during embryonic development, become aberrantly reactivated in cancer. They function coordinately to maintain self-renewal, block differentiation, and promote survival under stress conditions [34].

The Wnt/β-catenin pathway is arguably the most important regulator of CSC stemness in HCC. In the absence of Wnt signaling, β-catenin is continuously degraded by a destruction complex. Upon Wnt ligand binding to their receptors, this degradation is halted, allowing β-catenin to accumulate and translocate to the nucleus. In the nucleus, β-catenin interacts with T cell factor (TCF) and Lymphoid enhancer binding factor (LEF) transcription factors to activate target genes such as MYC, CCND1, and LGR5 [35]. In HCC, activating mutations in the β-catenin gene are found in about 20 to 30 percent of cases. Even in the absence of mutations, the pathway is frequently hyperactivated due to autocrine Wnt signaling or loss of negative regulators [36]. This hyperactivation maintains CSCs in an undifferentiated state and promotes chemoresistance. Several studies have demonstrated that blocking the Wnt β-catenin pathway reduces CSC self-renewal and restores sensitivity to multi kinase inhibitors [37].

The PI3K/Akt/mTOR pathway is another key regulator of CSC biology. This pathway is activated by growth factors and integrates signals from the TME [38,39]. It promotes cell survival, growth, and metabolism. In HCC CSCs, PI3K Akt signaling is often hyperactivated due to mutations in PIK3CA or loss of Phosphatase and tensin homolog (PTEN) function. This activation supports CSC maintenance by blocking apoptosis and enhancing protein synthesis. Inhibitors of this pathway have shown activity against CSCs in preclinical models [40].

The Notch pathway mediates cell-cell communication and controls cell fate decisions. It is activated when Notch receptors on one cell interact with Delta-like or Jagged ligands on a neighboring cell [41]. This interaction triggers proteolytic cleavage of the Notch receptor, releasing an intracellular domain that translocates to the nucleus and activates transcription of target genes, including Hairy and enhancer of split (HES) and Hairy/enhancer of split related with YRPW motif (HEY). In HCC, Notch signaling promotes CSC self-renewal and is associated with a poor prognosis [42].

The Hedgehog pathway is critical for embryonic patterning and tissue regeneration. In adult tissues, its activity is normally low. In HCC CSCs, however, Hedgehog signaling is frequently reactivated. The pathway is initiated by Sonic Hedgehog ligand binding to the Patched receptor, which relieves inhibition of Smoothened. Smoothened then activates GLI transcription factors, which drive expression of target genes involved in proliferation and survival [43].

Other pathways also contribute to CSC regulation. The NF-κB pathway mediates inflammatory signaling and promotes CSC survival under stress [44]. In conclusion, NF-κB plays a multifaceted role in HCC therapy, encompassing its complex molecular landscape, the development of therapeutic compounds targeting this pathway, and emerging nanomaterial-based strategies that improve drug delivery and overcome treatment resistance [45]. This work highlights the dual role of NF-κB in both promoting CSC stemness and orchestrating an immunosuppressive TME, making it an attractive yet challenging therapeutic target. The JAK/STAT pathway transmits signals from cytokines and growth factors and supports CSC self-renewal [46]. The TGF-β/SMAD pathway exerts context-dependent effects, promoting CSC maintenance in some settings while inducing differentiation in others. The net effect depends on the cellular context and the presence of other pathway activities [47]. From a systems biology perspective, these pathways form a highly interconnected network with extensive crosstalk and feedback loops. Blocking a single pathway is unlikely to fully eliminate CSCs, as compensatory activation of alternative pathways can sustain stemness (Fig. 2) [48]. Under therapeutic pressure, drug-sensitive CSC subpopulations may be eliminated, whereas resistant subpopulations can expand through clonal selection, leading to relapse. This underscores the need for combination strategies that target multiple pathways simultaneously and adapt over time.

### 2.3 Microenvironmental Support for CSCs

CSCs do not exist in isolation. They are embedded within a specialized niche known as the TME. This niche provides physical support and biochemical signals that maintain CSC stemness and protect CSCs from therapeutic attack. The microenvironment consists of various cell types, extracellular matrix components, and soluble factors [49].

Hypoxia, or low oxygen tension, is a common feature of solid tumors, including HCC. Hypoxic conditions activate hypoxia-inducible factors (HIFs), which then induce the expression of genes that promote CSC maintenance. HIF signaling enhances the expression of CSC markers such as CD133 and activates stemness-associated pathways including Wnt and Notch. CSCs tend to reside in hypoxic niches, which protect them from oxidative damage and chemotherapy [50].

TAMs are abundant immune cells within the HCC microenvironment. Most TAMs exhibit an M2-like polarization state, meaning they support tumor growth rather than attacking it. These macrophages secrete cytokines such as interleukin 6 and TGF beta, which activate STAT3 and NF-κB signaling in CSCs. This activation promotes CSC self-renewal and survival. Moreover, TAMs produce growth factors like epidermal growth factor that support CSC proliferation [51].

CAFs are another major cellular component of the microenvironment. These fibroblasts are activated by signals from tumor cells and acquire a pro-tumorigenic phenotype. CAFs secrete a variety of factors including hepatocyte growth factor, stromal derived factor 1, and various chemokines. These factors activate CSC signaling pathways and promote chemoresistance. CAFs also remodel the extracellular matrix, making it more favorable for CSC migration and invasion [52].

Exosomes are small vesicles secreted by both tumor cells and stromal cells. They carry proteins, lipids, and nucleic acids including microRNAs and long non coding RNAs. Exosomes from CAFs or TAMs can transfer stemness promoting signals to CSCs. For example, exosomes containing specific microRNAs have been shown to activate the Wnt/β-catenin pathway in recipient CSCs, enhancing their self-renewal capacity [53].

The close interaction between CSCs and their microenvironment creates a highly adaptive and resilient system. When one signaling pathway is blocked, CSCs can receive compensatory signals from the niche [54]. This is why targeting CSCs alone is often insufficient. Effective strategies must also disrupt the supportive microenvironment [55]. Notably, the epithelial-mesenchymal transition (EMT) plays a critical role in CSC maintenance and therapeutic resistance in HCC. A recent review by Alqurashi et al. (2023) provides a comprehensive nanotechnological perspective on capturing EMT targets in HCC, highlighting how nanoparticle-based delivery systems can be designed to simultaneously target EMT-associated CSC markers and overcome drug resistance [56]. This work underscores the potential of integrating nanomedicine with EMT-targeted strategies to enhance the efficacy of CSC-directed therapies.

Beyond responding to microenvironmental cues, HCC CSCs actively shape their immune landscape for survival. They secrete immunosuppressive factors that polarize TAMs toward the M2 phenotype, which in turn produce cytokines that reinforce CSC stemness, forming a positive feedback loop [57]. CSCs also express high levels of CD47 and PD-L1, inhibiting macrophage phagocytosis and T cell activity [58]. CSC-derived exosomes carry molecules that reprogram immune cells toward a pro-tumorigenic phenotype. This bidirectional crosstalk suggests that effective CSC eradication requires disrupting both CSC-intrinsic pathways and the immunosuppressive microenvironment. Triple combination strategies integrating a TKI, a CSC inhibitor, and an ICI may be necessary [59].

**Figure 2:**
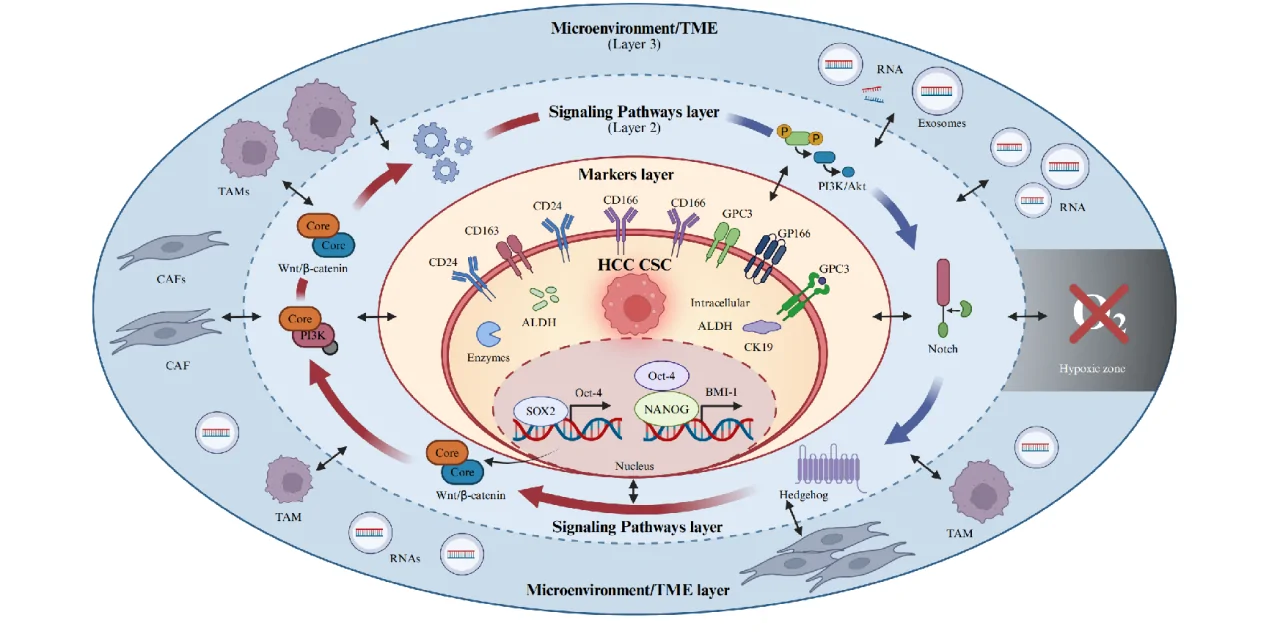
Signaling pathways regulating HCC CSC stemness. This schematic outlines the core signaling pathways (e.g., Wnt/β-catenin, PI3K/Akt) that regulate CSC stemness and survival in HCC. It highlights the central role of the Wnt/β-catenin pathway in maintaining the undifferentiated, drug-resistant state of CSCs. The network demonstrates how these pathways interact, creating a resilient system that sustains the CSC population. This figure was created using BioRender.

## 3 Mechanisms of Multikinase Inhibitor (MKI) Resistance

### 3.1 Multiple Mechanisms of TKI or MKI Resistance

Resistance to MKIs remains a major obstacle in the treatment of advanced HCC. Most patients who initially respond to drugs, such as sorafenib or Lenvatinib, eventually develop disease progression within six months [60]. This acquired resistance is complex and involves multiple interconnected mechanisms that allow tumor cells to survive and proliferate despite the presence of these drugs [61].

One well-established mechanism is the activation of alternative signaling pathways. When an MKI blocks one pathway, tumor cells can compensate by upregulating other pathways. For example, inhibition of the Raf/MEK/ERK pathway by sorafenib often leads to activation of the PI3K Akt pathway. Similarly, lenvatinib resistance has been linked to enhanced MET and EGFR signaling. These alternative pathways provide survival signals that bypass the drug effect [62].

Another important mechanism involves changes in drug transport and metabolism. Cancer cells can increase the expression of ATP-binding cassette (ABC) transporters such as ABCB1 and ABCG2 [63]. These transporters pump drugs out of the cell, reducing intracellular drug concentrations to subtherapeutic levels. This mechanism is particularly relevant in CSCs, which constitutively express high levels of these transporters [64].

Genetic and epigenetic alterations also contribute to TKI resistance. Secondary mutations in the drug targets can reduce drug binding affinity. Although such mutations are less common in HCC than in some other cancers, they have been reported. Epigenetic changes, such as DNA methylation and histone modification, can also alter gene expression patterns to favor survival under drug pressure [65].

However, a growing body of evidence points to a more fundamental mechanism of resistance. The conventional view of TKI resistance focuses on how bulk tumor cells adapt to drug exposure. Specifically, this view overlooks the fact that tumors are not homogeneous [66]. They contain a small subpopulation of CSCs that are intrinsically resistant to many therapies. These CSCs survive TKI treatment and subsequently repopulate the tumor, leading to relapse with acquired resistance [67].

### 3.2 Empirical Links between CSC Markers and TKI Resistance

Numerous studies have established strong associations between CSC marker expression and resistance to MKIs in HCC. These associations have been observed in cell lines, animal models, and clinical samples [68].

The CD133/Myosin heavy chain 9 (MYH9) β-catenin axis is one well characterized mechanism. CD133, a cell surface marker of HCC CSCs, physically interacts with MYH9, a non-muscle myosin. This interaction stabilizes active β-catenin by preventing its degradation. The accumulated β-catenin then translocates to the nucleus and drives the expression of genes involved in self-renewal and drug resistance. In sorafenib-resistant HCC cell lines, CD133 expression is significantly upregulated. Silencing CD133 restores sensitivity to sorafenib. Clinical studies have shown that HCC patients with high CD133 expression have poorer responses to sorafenib treatment and shorter progression-free survival.

Caveolin 1 (CAV1)-mediated tumor dormancy represents another important resistance mechanism. CAV1 is a scaffolding protein that is highly expressed in a subset of HCC CSCs. High CAV1 expression maintains CSCs in a dormant state through the STAT3 P70S6K signaling pathway (Fig. 3). Dormant CSCs are slowly cycling or non cycling, making them intrinsically resistant to TKIs that target rapidly proliferating cells. These dormant cells survive TKI treatment and can be reactivated later to drive tumor recurrence. In TKI non responder patients, CAV1-positive cell subpopulations are significantly enriched.

EpCAM-positive CSCs have also been linked to TKI resistance. EpCAM is a marker of epithelial-like CSCs that are highly proliferative. These cells are not dormant but actively dividing. They resist TKI treatment through high expression of ABC transporters and activation of the Wnt/β-catenin pathway. In lenvatinib resistant models, EpCAM positive cells are expanded, and blocking the Wnt pathway restores lenvatinib sensitivity [69].

CD13 marks quiescent CSCs that are particularly resistant to conventional TKIs. These cells are in a slow-cycling state and express high levels of survival proteins. They can persist through months of TKI treatment and serve as a reservoir for tumor recurrence. CD13-positive CSCs have been identified in both sorafenib-resistant and Lenvatinib-resistant models [70].

The emerging link between MASLD-associated HCC and CSC-mediated resistance deserves special attention. In MASLD-associated HCC, CD133-positive CSCs are significantly enriched compared to viral HCC [71]. These cells show higher expression of resistance-related genes and are less responsive to sorafenib [72]. This finding suggests that the etiology of HCC influences CSC composition and thereby affects treatment outcomes.

Taken together, these empirical findings support a unified view [73]. CSCs, marked by various surface markers and driven by distinct molecular pathways, are a major cellular source of TKI resistance. Targeting these CSCs, rather than just the bulk tumor, may overcome resistance and improve patient outcomes.

An important dimension of CSC heterogeneity is the influence of underlying liver disease etiology. HCC arising from different etiologies—MASLD, HBV, and HCV—exhibits distinct patterns of CSC marker expression and metabolic reprogramming. In MASLD-associated HCC, CD133-positive CSCs are significantly enriched compared to viral hepatitis-related HCC, driven by the lipotoxic and inflammatory microenvironment that activates specific signaling pathways [74]. MASLD-derived CSCs also show enhanced lipid metabolism and oxidative stress tolerance, with upregulation of certain lipid biosynthesis enzymes, making them vulnerable to inhibitors targeting the related metabolic axis. In contrast, HBV-related HCC is characterized by a higher proportion of EpCAM-positive CSCs, driven by HBV X protein activation of the Wnt/β-catenin pathway. HCV-related HCC shows enrichment of CD44-positive CSCs, possibly due to HCV core protein-induced TGF-β signaling. These etiology-dependent differences suggest that patient stratification by disease etiology could optimize CSC-targeted therapies, moving toward a personalized medicine approach for HCC [75].

**Figure 3:**
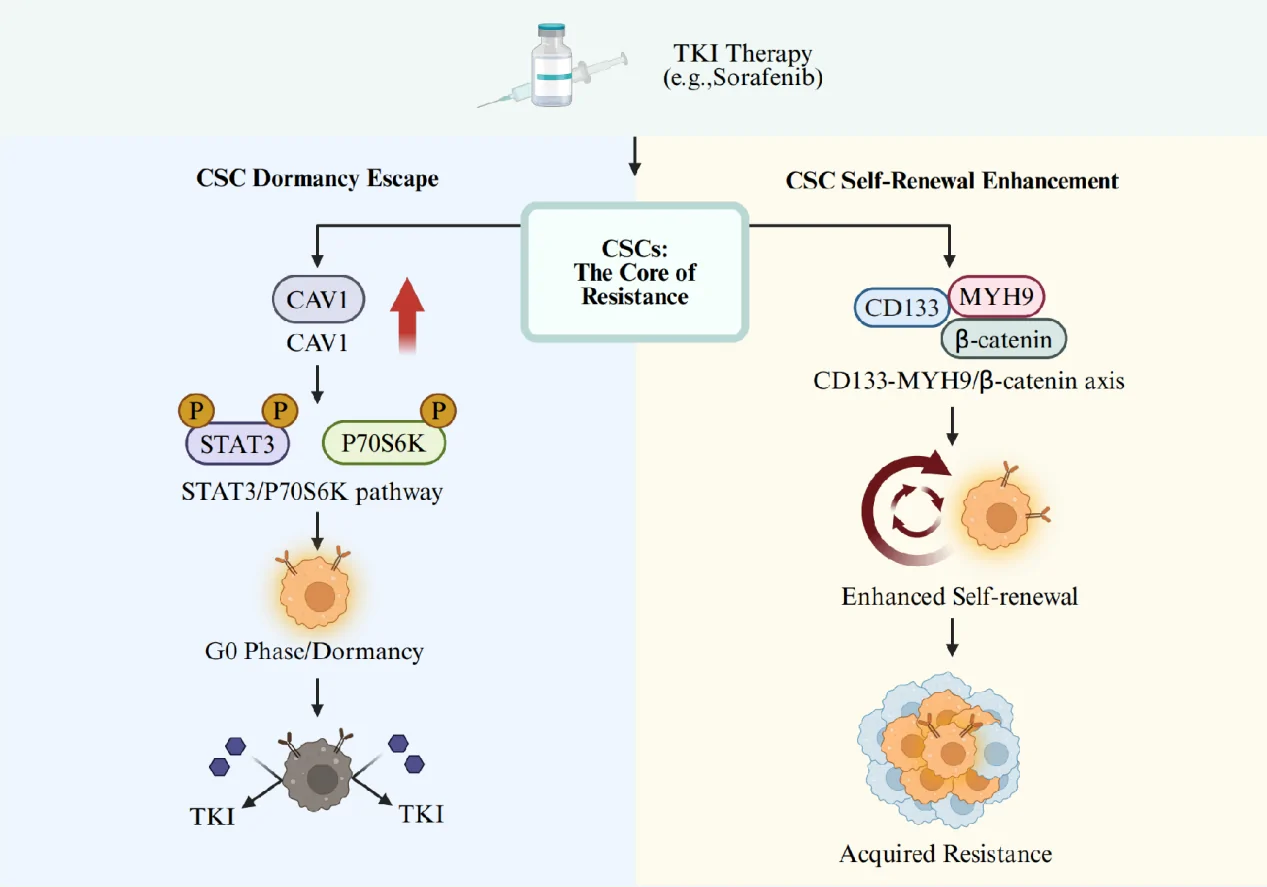
Molecular mechanisms of tyrosine kinase inhibitor (TKI) drug resistance. This figure explains how CSCs cause resistance to drugs like sorafenib through specific mechanisms. It illustrates two key processes: activation of the (Cluster of Differentiation 133) CD133-Myosin heavy chain 9 (MYH9)-β-catenin survival axis and entry into a Caveolin 1 (CAV1)-mediated dormant state. These mechanisms allow CSCs to survive treatment and later repopulate the tumor, driving therapeutic failure. This figure was created using BioRender.

## 4 Small Molecule Inhibitors Targeting CSCs

### 4.1 Inhibitors Targeting the Wnt/β-Catenin Pathway

The Wnt/β-catenin pathway is a central regulator of CSC stemness in HCC. Hyperactivation of this pathway maintains CSCs in an undifferentiated state and drives resistance to conventional therapies. Therefore, pharmacological inhibition of this pathway represents a promising strategy to eliminate CSCs and overcome treatment resistance.

C504244 is a diarylpyrimidine guanidine derivative that has demonstrated potent activity against HCC CSCs. Unlike many other Wnt inhibitors that target the ligand-receptor interaction or β-catenin degradation, C504244 acts by directly disrupting the formation of the β-catenin/Transcription factor 4 (TCF4) transcription factor complex. This complex is essential for the transcriptional activity of β-catenin [76]. By blocking its formation, C504244 effectively suppresses the expression of Wnt target genes involved in self-renewal and proliferation. In preclinical studies, C504244 significantly reduced the sphere-forming capacity of HCC cells, a functional measure of CSC abundance. It also inhibited cell proliferation and migration. More importantly, C504244 reversed lenvatinib resistance in resistant HCC cell lines and xenograft models. When combined with lenvatinib, the combination regimen showed superior antitumor activity compared to either drug alone [76]. However, the clinical translation of C504244 faces several challenges. Its oral bioavailability and pharmacokinetic profile in humans remain unknown, as all studies to date have been preclinical. Systemic inhibition of Wnt/β-catenin signaling may cause on-target toxicity in intestinal crypts and bone marrow, thereby limiting the therapeutic window [76]. Further studies are needed to evaluate safety, dosing, and tumor selectivity before clinical trials can proceed.

Inhibitor of catenin 2 (IC 2) is another small molecule inhibitor that targets β-catenin transcriptional activity. It was identified through a high-throughput screen for compounds that block the interaction between β-catenin and its coactivators [77]. IC 2 reduces the expression of CSC markers and inhibits the self-renewal of CSCs. Although less extensively studied than C504244 in HCC, IC 2 has shown promising activity in preclinical models and warrants further investigation [78].

Other Wnt pathway inhibitors have also been evaluated in HCC CSC models [79]. These include small molecules targeting upstream components of the pathway such as porcupine inhibitors that block Wnt ligand secretion, and tankyrase inhibitors that stabilize the β-catenin destruction complex. However, many of these agents have shown off target effects or limited activity against CSCs, highlighting the importance of targeting the pathway at the level of the β-catenin transcription complex [80].

### 4.2 Strategies Targeting CSC Cell Surface Markers

Cell surface markers expressed on CSCs provide attractive targets for therapeutic intervention. Unlike intracellular targets, cell surface markers are accessible to antibodies and certain small molecules. Several approaches have been developed to target these markers [81].

4 methylumbelliferone (4 MU) is a small molecule inhibitor of hyaluronic acid synthesis. Hyaluronic acid, a major component of the extracellular matrix, is involved in CSC maintenance [82]. 4 MU treatment downregulates the expression of multiple CSC markers including CD133, CD90, EpCAM, CD44, and CD13 [83]. This broad effect on CSC markers is unusual for a single agent and suggests that 4 MU may disrupt the CSC niche rather than directly inhibiting a specific marker. In preclinical models, 4 MU treatment reduced the proportion of CSCs and suppressed tumor growth [84]. Notably, combining 4 MU with Interleukin 12 (IL 12) gene therapy induced M1 polarization of TAMs and further prolonged survival in tumor bearing mice [85]. This combination strategy simultaneously targets CSCs and the immunosuppressive microenvironment [86]. Despite these promising results, several barriers hinder clinical translation. 4-MU is a relatively weak inhibitor of its target, requiring high doses to achieve therapeutic effects, which may raise concerns about off-target toxicity [87]. Furthermore, IL-12 gene therapy has historically been associated with severe systemic inflammation in clinical trials, necessitating careful dose optimization and delivery strategies to avoid adverse immune reactions [88]. The lack of validated biomarkers to monitor the *in vivo* activity of this combination also complicates clinical development.

Bestatin is a CD13 inhibitor that targets a distinct CSC subpopulation. CD13 positive CSCs are characterized by their quiescent or slow cycling state. These cells are particularly resistant to conventional TKIs because they are not actively dividing. Bestatin eliminates these quiescent CSCs by inducing apoptosis specifically in CD13-positive cells [89]. In preclinical studies, bestatin treatment reduced tumor recurrence after TKI therapy by depleting the dormant CSC reservoir. This approach is complementary to TKIs, which kill rapidly dividing bulk tumor cells [90]. However, the clinical utility of bestatin is limited by its modest potency as a single agent and an incomplete understanding of CD13 expression dynamics in human HCC [45]. It remains unclear whether bestatin can effectively reach quiescent CSCs within their hypoxic niches, as drug penetration may be suboptimal. Furthermore, the long-term safety of depleting quiescent stem cell populations in the liver, which may be required for tissue regeneration, warrants careful investigation.

Antibody based approaches targeting CSC surface markers have also been developed. Monoclonal antibodies targeting CD133, EpCAM, and CD44 have shown activity in preclinical HCC models. These antibodies can directly induce CSC death through antibody dependent cellular cytotoxicity or complement dependent cytotoxicity [91]. Some antibodies are conjugated to toxins or drugs to create antibody drug conjugates that deliver cytotoxic payloads specifically to CSCs. However, antibody-based therapies have limitations, including high cost, the need for intravenous administration, and potential immunogenicity [92].

### 4.3 Natural Products Modulating Metabolism and Stemness

Natural products have long been a rich source of anticancer agents. Several natural compounds have been identified that specifically target CSC metabolism and stemness in HCC [93].

Curcumin is a polyphenol derived from turmeric. It has been extensively studied for its anticancer properties. In HCC CSC models, curcumin inhibits self-renewal and induces differentiation [94]. It achieves these effects by downregulating the expression of pluripotency transcription factors, such as SOX2 and OCT4 [95]. Curcumin also reduces the activity of the Wnt/β-catenin pathway and the PI3K/Akt pathway [96]. Despite its potent activity in cell culture, curcumin has poor bioavailability, which has limited its clinical development. Formulation strategies such as nanoparticles are being explored to overcome this limitation [97].

Salinomycin is an ionophore antibiotic that was originally used as a veterinary drug. It gained attention in cancer research when a high throughput screen identified it as a selective killer of breast CSCs. Subsequent studies have shown that salinomycin also eliminates CSCs in HCC [67]. It works by disrupting ion homeostasis and inducing oxidative stress in CSCs. Salinomycin treatment reduces the ALDH-positive cell population and inhibits sphere formation. However, its toxicity to normal cells remains a concern [98].

Berberine is an alkaloid found in several medicinal herbs. It has been shown to reduce CSC self-renewal by inhibiting the Hedgehog signaling pathway [99]. Berberine treatment decreases the expression of CSC markers and sensitizes HCC cells to sorafenib [100].

Paris saponins I and VII are steroidal saponins extracted from the medicinal plant Paris polyphylla [101]. These compounds have shown selective toxicity against ALDH positive CSCs. They reduce the proportion of ALDH positive cells and inhibit tumor initiation in xenograft models. The mechanism involves the induction of apoptosis through activation of the mitochondrial pathway [102].

Betulinic acid is a pentacyclic triterpene found in birch bark. It inhibits CSC self-renewal and induces differentiation. Betulinic acid also synergizes with sorafenib to eliminate CSCs and suppress tumor growth [103].

### 4.4 Targeting Kinases Involved in CSC Regulation

Kinases are attractive drug targets because they are enzymatically active and can be inhibited by small molecules. Several kinases play critical roles in CSC maintenance and represent therapeutic opportunities [104].

OSU-CG5 is a small molecule inhibitor of Pyruvate dehydrogenase kinase 1 (PDK1), a kinase activated by the PI3K/Akt pathway, which inhibits pyruvate dehydrogenase (PDH). PDK1 is overexpressed in HCC CSCs and contributes to their survival and self-renewal [105]. OSU-CG5 treatment reduces the CSC population and suppresses sphere formation [106]. It also sensitizes CSCs to sorafenib by blocking the compensatory activation of the PI3K/Akt pathway that occurs during TKI treatment [107]. Despite encouraging preclinical findings, OSU-CG5 faces translational hurdles. Systemic PDK1 inhibition may cause metabolic disturbances, especially hyperglycemia and insulin resistance, concerning in HCC patients with liver dysfunction. Its pharmacokinetic properties remain poorly characterized. The lack of predictive biomarkers further complicates trial design. LDN193189 is a Bone morphogenetic protein (BMP) inhibitor that has shown activity against HCC CSCs. BMP signaling, which is part of the TGF beta superfamily, supports CSC maintenance in some contexts. LDN193189 blocks BMP signaling and reduces CSC stemness. However, the role of BMP signaling in HCC CSCs is complex and context dependent, requiring careful patient selection for this approach [108].

Other kinase inhibitors targeting CSC-related kinases are in development. These include inhibitors of Focal adhesion kinase (FAK), which is involved in CSC migration and survival, and inhibitors of AXL, which is upregulated in drug resistant CSCs. The emerging understanding of CSC kinase signaling is revealing new targets for therapeutic intervention (Fig. 4) [109] (Table 2).

**Table 2:** Small-Molecule compounds targeting CSCs in hepatocellular carcinoma.

Compound	Target/Pathway	Mechanism of Action	Effect on CSCs	Research Stage	Citation
C504244	β-catenin/TCF4	Disrupts transcription complex formation	Inhibits sphere formation and migration, reverses resistance	Preclinical	[76,110]
4 MU	Hyaluronic acid synthesis	Downregulates CD133, CD90, and EpCAM expression	Reduces CSC proportion	Preclinical	[111]
Bestatin	CD13	Eliminates quiescent CSCs	Clears dormant CSCs	Preclinical	[112]
Paris saponins I and VII	ALDH	Reduces proportion of ALDH positive cells	Attenuates CSC stemness	Preclinical	[113]
OSU-CG5	PDK1	Inhibits PDK1 kinase activity	Attenuates CSC self-renewal	Preclinical	[114]
CL26	AGPAT4	Inhibits mTOR signaling	Synergizes with sorafenib for enhanced efficacy	Preclinical	[115]

Abb: CSC→Cancer stem cell, TCF4→Transcription factor 4, 4 MU→4 methylumbelliferone, CD→Cluster of Differentiation, ALDH→Aldehyde dehydrogenase, PDK1→Pyruvate dehydrogenase kinase 1, AGPAT4→1 acylglycerol 3 phosphate O acyltransferase 4.

**Figure 4:**
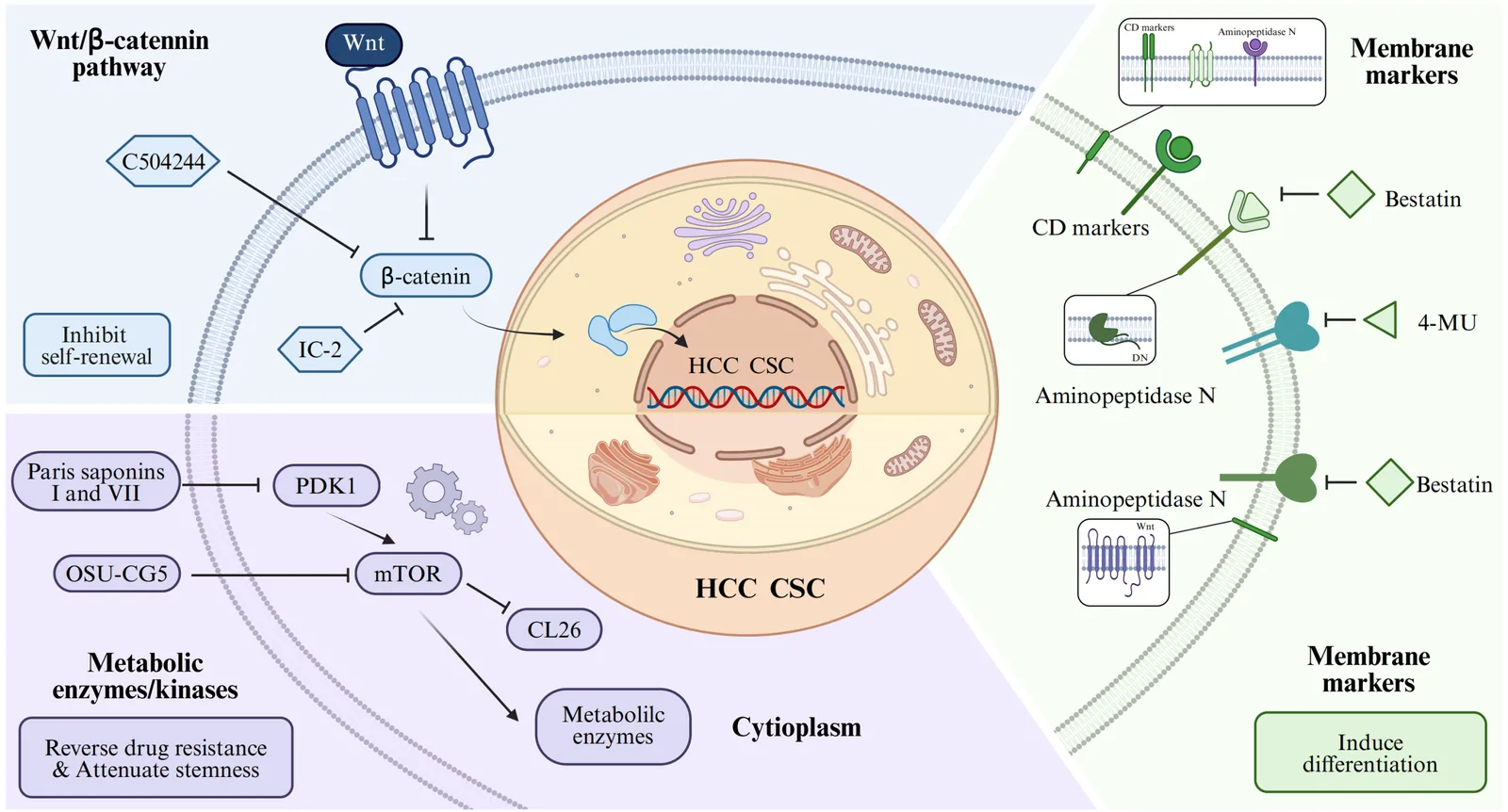
Small molecule inhibitors targeting CSCs. This figure summarizes preclinical small-molecule inhibitors designed to target HCC CSCs. Examples include C504244 (blocks Wnt signaling), 4-methylumbelliferone (downregulates CSC markers), and bestatin (targets dormant CSCs). These agents show promise in reducing CSC self-renewal and overcoming drug resistance in laboratory models. This figure was created using BioRender.

## 5 Emerging Therapeutic Strategies: Combination and Precision Delivery

### 5.1 Synergistic Strategies Combining TKIs with CSC Inhibitors

The recognition of CSCs as a major source of TKI resistance has driven the development of combination strategies that simultaneously target the bulk tumor and the CSC reservoir. These combinations are designed to achieve what neither agent can accomplish alone: durable tumor control and the prevention of relapse.

One promising approach combines sorafenib with nanoparticles loaded with CD133 small interfering RNA (siRNA). CD133 is a well-established CSC marker in HCC, and its expression is enriched in MASLD associated HCC, a subtype that responds poorly to many therapies (Fig. 5). In MASLD HCC models, CD133 positive CSCs are responsible for sorafenib resistance. By delivering siRNA that specifically silences CD133 expression, these nanoparticles effectively reduce the CSC population. When combined with sorafenib, the dual treatment significantly enhances antitumor efficacy compared to either agent alone. The key mechanism involves downregulation of CD133, which disrupts the CD133 MYH9 β-catenin axis and restores sensitivity to sorafenib [116].

Another important combination is lenvatinib plus C504244. Lenvatinib is a first-line TKI that inhibits multiple receptor tyrosine kinases including Vascular endothelial growth factor receptor (VEGFR), Fibroblast growth factor receptor (FGFR), and Platelet-derived growth factor receptor (PDGFR). However, resistance inevitably develops, and β-catenin activation is a major mechanism of acquired resistance to lenvatinib. C504244 directly targets the β-catenin–TCF4 transcription complex, thereby overcoming this resistance. In Lenvatinib-resistant HCC cell lines and xenograft models, the combination of lenvatinib and C504244 suppresses tumor growth more effectively than lenvatinib alone. The key mechanism is inhibition of β-catenin transcriptional activity, which reduces CSC self-renewal and restores lenvatinib sensitivity.

The combination of sorafenib with siCAV1 or miR 7 targets the CAV1-mediated dormancy mechanism. CAV1-positive CSCs are dormant and therefore resistant to sorafenib, which targets rapidly dividing cells [117]. By silencing CAV1 expression or delivering miR 7, a microRNA that targets CAV1, these dormant CSCs are reactivated and become sensitive to sorafenib. This strategy effectively converts a resistant cell population into a sensitive one. In HCC cell models, this combination treatment reverses CAV1 driven resistance and eliminates the dormant CSC reservoir [118].

The combination of sorafenib with CL26 targets the AGPAT4-mTOR axis. AGPAT4 is a metabolic enzyme that is upregulated in CSCs and promotes mTOR signaling. CL26 is a novel small-molecule inhibitor of AGPAT4. When used alone, CL26 exhibits modest antitumor activity. However, when combined with sorafenib, the two agents act synergistically to suppress tumor growth in HCC cell lines and xenograft models. The key mechanism involves the inhibition of the AGPAT4-mTOR axis, which reduces CSC self-renewal and proliferation. (Table 3).

**Table 3:** Summary of synergistic strategies combining TKIs with CSC inhibitors.

Combination Regimen	Model System	Synergistic Effect	Key Mechanism
Sorafenib plus CD133 siRNA nanoparticles	MASLD HCC model	Significantly enhances antitumor efficacy	Downregulates CD133, reverses drug resistance
Lenvatinib plus C504244	Resistant HCC cells and nude mice	Overcomes β-catenin mediated resistance	Inhibits β-catenin transcriptional activity
Sorafenib plus siCAV1 or miR 7	HCC cells	Reverses CAV1 driven resistance	Releases CSC from dormant state
Sorafenib plus CL26	HCC cells and tumor bearing mice	Synergistically suppresses tumor growth	Inhibits AGPAT4 mTOR axis

Abb: CD→Cluster of Differentiation, MASLD→Metabolic dysfunction associated steatotic liver disease, CAV1→Caveolin 1, HCC→Hepatocellular carcinoma, CSC→Cancer stem cell, AGPAT4 1 acylglycerol 3 phosphate O acyltransferase 4.

### 5.2 The Promise of siRNA Targeting CSCs

RNA interference using siRNA offers a powerful approach to silence genes that are essential for CSC maintenance. Unlike small-molecule inhibitors, which often have off-target effects and may not completely block protein function, siRNA can achieve highly specific and potent gene silencing.

Several CSC-related genes have been targeted by siRNA in preclinical HCC models. CD133 siRNA reduces the expression of this CSC marker and eliminates the CD133-positive CSC population. EpCAM siRNA similarly targets EpCAM-positive CSCs. β-catenin siRNA silences the central regulator of the Wnt pathway and reduces CSC self-renewal.

Despite its promise, siRNA therapy faces major delivery challenges. Naked siRNA is rapidly degraded in the bloodstream by nucleases. It does not efficiently enter target cells due to its negative charge and large molecular weight. It can also trigger off-target immune responses. To overcome these challenges, various nanocarriers have been developed. These include lipid-based nanoparticles, polymeric nanoparticles, and exosomes. Lipid nanoparticles are the most advanced platform and have been successfully used to deliver siRNA to the liver. In fact, the first Food and Drug Administration (FDA)-approved siRNA therapeutic, patisiran, utilizes lipid nanoparticle technology. For HCC CSC targeting, nanocarriers can be surface-modified with antibodies or aptamers that recognize CSC markers such as CD133 or EpCAM. This active targeting approach enhances siRNA delivery specifically to CSCs [119].

The combination of siRNA with TKIs represents a rational therapeutic strategy. The TKI kills rapidly dividing bulk tumor cells, while the siRNA eliminates the CSC population. This dual approach has shown promise in preclinical models and warrants further investigation.

### 5.3 Nanomedicine for CSC Targeted Delivery

Nanomedicine offers unique advantages for CSC-targeted therapy. Nanoparticles can be designed to carry multiple drugs, protect them from degradation, release them in a controlled manner, and target specific cell populations.

The TME presents multiple barriers to drug delivery. Poor vascularization, high interstitial pressure, and a dense extracellular matrix limit the penetration of therapeutic agents. Nanoparticles can overcome some of these barriers through the enhanced permeability and retention effect, which allows nanoparticles to accumulate in tumors due to leaky blood vessels and impaired lymphatic drainage. However, this passive targeting strategy is not sufficient to deliver drugs specifically to CSCs, which often reside in hypoxic niches deep within the tumor.

Active targeting strategies address this limitation. Nanoparticles are surface-modified with targeting ligands that bind to CSC markers. For example, anti-CD133 antibodies or aptamers can be conjugated to the nanoparticle surface. These modified nanoparticles preferentially bind to CD133-positive CSCs and are internalized through receptor-mediated endocytosis. Similarly, anti-EpCAM and anti-CD44 targeting ligands have been used to deliver drugs to HCC CSCs.

Co-delivery of multiple agents is another advantage of nanomedicine. A single nanoparticle can be loaded with both a TKI and a CSC inhibitor, ensuring that both drugs reach the same target cells simultaneously. This co delivery approach is particularly important for combination strategies where both agents are needed to achieve the desired effect. For example, nanoparticles co-delivering sorafenib and CD133 siRNA have shown superior efficacy compared to either agent alone or the combination of free drugs.

Other nanocarrier systems, including polymeric micelles, dendrimers, and exosomes are also being explored. Exosomes are particularly interesting because they are naturally occurring nanoparticles that mediate intercellular communication. They can be harvested from cells and loaded with therapeutic cargo. Exosomes derived from immune cells or stem cells have demonstrated intrinsic targeting properties that may be exploited for drug delivery to CSCs.

### 5.4 New Metabolic Target: AGPAT4

Metabolic reprogramming is increasingly recognized as a hallmark of CSCs. Unlike bulk tumor cells, which rely heavily on glycolysis, CSCs exhibit flexible metabolic profiles that allow them to adapt to changing environmental conditions. This metabolic plasticity provides survival advantages but also creates vulnerabilities that can be therapeutically exploited.

AGPAT4 is an enzyme involved in lipid biosynthesis. It catalyzes the conversion of lysophosphatidic acid to phosphatidic acid, a key step in the synthesis of triglycerides and phospholipids. In HCC CSCs, AGPAT4 is highly expressed compared to non-CSCs and normal hepatocytes. This elevated expression promotes mTOR signaling, which supports CSC self-renewal and survival [120].

CL26 is a novel small-molecule inhibitor of AGPAT4 that was identified through structure-based drug design. It binds to the active site of AGPAT4 and inhibits its enzymatic activity. In preclinical studies, CL26 treatment reduces mTOR signaling and suppresses CSC self renewal [69]. When used alone, CL26 exhibits modest antitumor activity. However, when combined with sorafenib, the two agents show synergistic effects. This combination treatment significantly suppresses tumor growth in HCC cell lines and xenograft mouse models. Importantly, CL26 has a favorable safety profile with low toxicity to normal tissues [121]. Caution is warranted for CL26's translation, as its low toxicity reflects limited exposure rather than target selectivity, and its modest activity and undefined combination dosing suggest it is primarily a sensitizer, while uncharacterized AGPAT4 expression in HCC raises concerns about target prevalence. The discovery of the AGPAT4-mTOR axis as a therapeutic target in HCC CSCs opens new avenues for drug development. Other metabolic enzymes involved in lipid synthesis, glutamine metabolism, and mitochondrial function may also represent viable targets for CSC-directed therapy. Further research is needed to identify the most vulnerable metabolic nodes in HCC CSCs and to develop inhibitors that can be safely used in patients [122].

**Figure 5:**
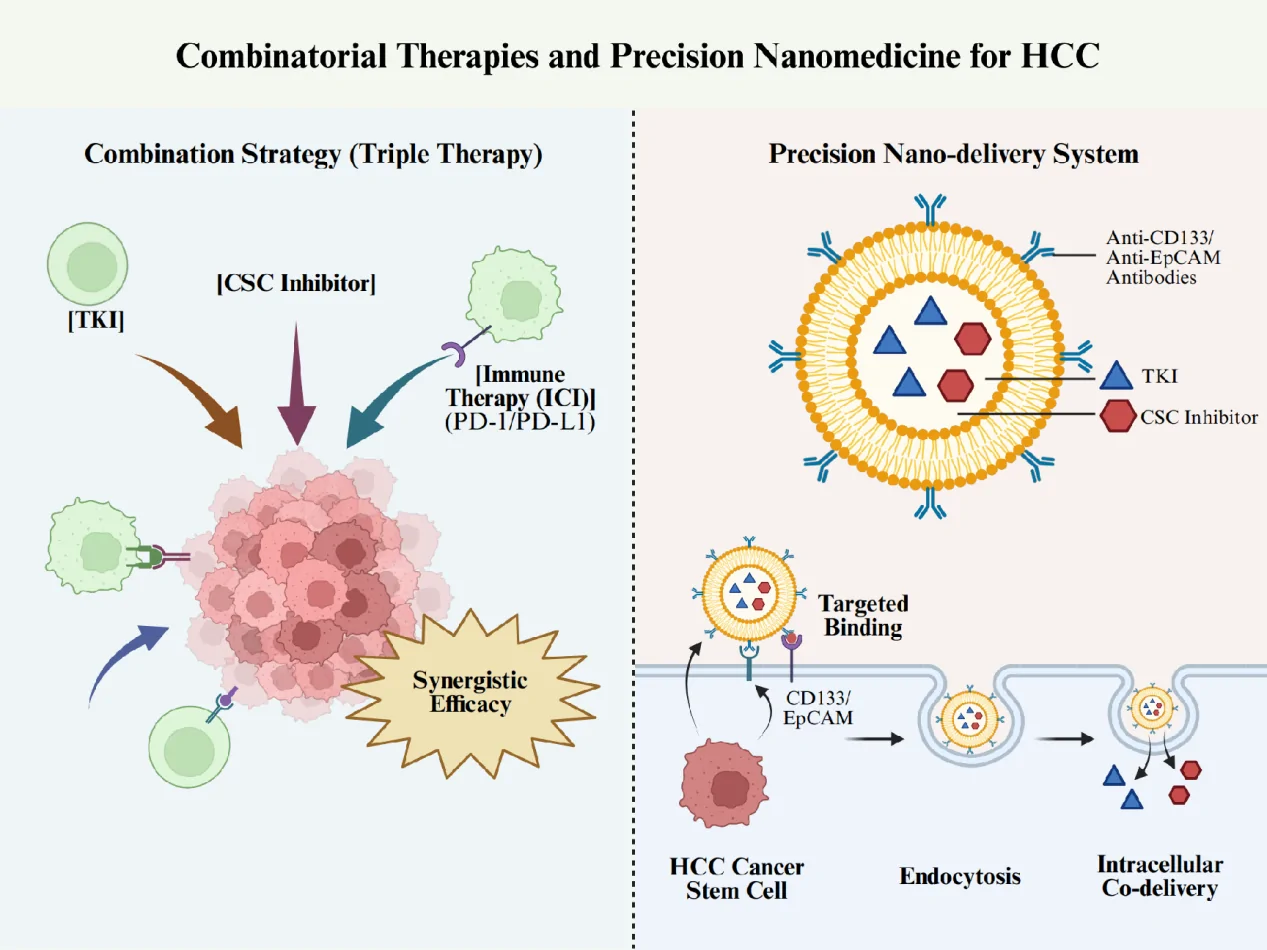
Synergistic strategies and Nano-delivery systems. This diagram presents the use of nanoparticles to deliver drugs specifically to CSCs, often by attaching targeting ligands like anti-CD133 antibodies. It highlights synergistic strategies, such as co-delivering a TKI (e.g., sorafenib) with a CSC inhibitor (e.g., siRNA) in a single nanoparticle. This approach aims to simultaneously kill bulk tumor cells and the CSC reservoir, representing a promising direction for overcoming resistance. This figure was created using BioRender.

## 6 Conclusion and Perspectives

### 6.1 Integration of Core Concepts

This review has traced the evolution of targeted therapy for HCC, from conventional multikinase inhibition to emerging CSC eradication strategies. The central argument is straightforward: MKIs such as sorafenib and lenvatinib can suppress tumor growth and prolong survival, but they cannot eliminate the root cause of tumor initiation, relapse, and metastasis, which is the CSC population [123].

HCC CSCs are characterized by specific cell surface markers, intracellular enzymes, and transcription factors that maintain their self-renewal capacity. They are regulated by a network of developmentally conserved signaling pathways, including Wnt/β-catenin, PI3K/Akt/mTOR, and Notch [124]. Their survival depends not only on intrinsic pathways but also on supportive cues from the TME, including hypoxia, TAMs, and CAFs [125].

Resistance to MKIs is largely driven by CSCs through mechanisms such as CAV1-mediated dormancy and CD133/MYH9/β-catenin axis activation. Targeting these mechanisms with small-molecule inhibitors, such as C504244, 4 MU, bestatin, OSU-CG5, and CL26, has shown promise in preclinical models. The most effective approach may be combination strategies that pair TKIs with CSC inhibitors, sometimes delivered via nanoparticle systems for precision targeting. The emergence of metabolic targets, such as AGPAT4, further expands the therapeutic toolbox.

### 6.2 Current Bottlenecks

Despite significant progress, several bottlenecks hinder the translation of CSC-targeted therapies into clinical practice. First, most small-molecule inhibitors discussed in this review remain at the preclinical stage [126]. Their efficacy, safety, pharmacokinetics, and pharmacodynamics have not been validated in clinical trials. Notably, the history of Wnt pathway inhibitors in oncology is marked by clinical failures. Previous inhibitors caused on-target toxicities—such as bone fragility and gastrointestinal adverse effects—that limited their therapeutic window [116]. These failures serve as cautionary tales for β-catenin inhibitors like C504244, highlighting the need for careful dose optimization and tumor-selective delivery. Second, CSC heterogeneity poses a major challenge. HCC tumors contain multiple CSC subpopulations, marked by different surface markers and driven by different signaling pathways. Targeting one CSC subset may leave another intact, thereby allowing eventual relapse [127]. For instance, a therapy that effectively eliminates EpCAM-positive CSCs may still permit CD90-positive mesenchymal CSCs to thrive and drive metastasis, highlighting the need for combinatorial targeting of multiple CSC subsets [128]. Third, the TME is highly adaptive. When one pathway is blocked, CSCs receive compensatory signals from the niche. Fourth, reliable biomarkers for patient stratification and treatment monitoring are lacking. Without such biomarkers, it is difficult to identify which patients are most likely to benefit from CSC-targeted therapy [129].

### 6.3 Future Directions

Several promising future directions are worth pursuing. First, combination therapy is the inevitable path forward. Triple combinations of a TKI, a CSC inhibitor, and an immunotherapy agent, such as an ICI, may achieve the most durable responses. Second, precision delivery systems, including nanoparticles and exosomes, will be essential for clinical translation. These technologies can improve drug bioavailability, reduce off-target toxicity, and enable active targeting of CSCs. Third, biomarker development must be prioritized. CSC marker expression levels, pathway activity signatures, and circulating tumor cell analyses could guide treatment selection and monitor response [130]. Fourth, the metabolic vulnerabilities of CSCs warrant deeper exploration. AGPAT4 and other metabolic enzymes may represent druggable targets with favorable safety profiles. Finally, the etiology of HCC influences CSC composition and drug sensitivity. Future trials should stratify patients by disease etiology to optimize treatment allocation [131].

The shift from multikinase inhibition to CSC eradication represents a true paradigm shift. With continued research and development, this strategy holds the promise of transforming HCC from a relapsing and often fatal disease into a manageable, or even curable, condition.

CSC complexity necessitates multi-omics approaches. Integrating single-cell/spatial transcriptomics, proteomics, and metabolomics can help characterize the CSC landscape. CD133-positive CSCs are enriched at the invasive front, EpCAM-positive CSCs in the tumor core, and CD90-positive CSCs at the tumor-stroma interface [132]. CSCs at the invasive edge are more exposed to hypoxia and immune cells, driving plasticity and resistance—a finding that can guide spatially targeted therapies.

The neural microenvironment is an emerging area of interest. The liver is innervated by autonomic nerves; neurotransmitters activate receptors on CSCs, promoting self-renewal and the expression of anti-apoptotic genes. The neural microenvironment also indirectly supports CSCs by promoting angiogenesis and modulating immune cell infiltration. Targeted neural intervention could disrupt the CSC niche, overcome drug resistance, and inhibit HCC recurrence [133].

## Data Availability

No new data were generated or analyzed in this review article. All information presented is derived from previously published studies and publicly available sources. Data sharing is not applicable to this article.

## Abbreviation

4 MU	4 methylumbelliferone
ABC	ATP-binding cassette
AGPAT4	1 acylglycerol 3 phosphate O acyltransferase 4
ALDH	Aldehyde dehydrogenase
BMI 1	B cell specific Moloney murine leukemia virus integration site 1
BMP	Bone morphogenetic protein
CAF	Cancer associated fibroblast
CAV1	Caveolin 1
CSC	Cancer stem cell
EGFR	Epidermal growth factor receptor
EpCAM	Epithelial cell adhesion molecule
FAK	Focal adhesion kinase
FDA	Food and Drug Administration
FGFR	Fibroblast growth factor receptor
GPC3	Glypican 3
HCC	Hepatocellular carcinoma
HES	Hairy and enhancer of split
HEY	Hairy/enhancer of split related with YRPW motif
HIF	Hypoxia inducible factor
IC 2	Inhibitor of catenin 2
ICI	Immune checkpoint inhibitor
IL 12	Interleukin 12
JAK	Janus kinase
KLF4	Kruppel like factor 4
LEF	Lymphoid enhancer binding factor
LGR5	Leucine rich repeat containing G protein coupled receptor 5
MASLD	Metabolic dysfunction associated steatotic liver disease
MET	Mesenchymal epithelial transition factor
MKI	Multikinase inhibitor
mTOR	Mammalian target of rapamycin
MYC	Myelocytomatosis oncogene
MYH9	Myosin heavy chain 9
NANOG	Homeobox protein NANOG
NF-κB	Nuclear factor kappa B
OCT4	Octamer binding transcription factor 4
OS	Overall survival
PD 1	Programmed cell death protein 1
PDGFR	Platelet derived growth factor receptor
PDK1	Pyruvate dehydrogenase kinase 1
PD L1	Programmed death ligand 1
PI3K	Phosphoinositide 3 kinase
PTEN	Phosphatase and tensin homolog
SALL4	Spalt like transcription factor 4
siRNA	Small interfering RNA
SOX2	SRY box transcription factor 2
STAT	Signal transducer and activator of transcription
TAM	Tumor associated macrophage
TCF	T cell factor
TCF4	Transcription factor 4
TGF beta	Transforming growth factor beta
TKI	Tyrosine kinase inhibitor
TME	Tumor microenvironment
Treg	Regulatory T cell
VEGF	Vascular endothelial growth factor
VEGFR	Vascular endothelial growth factor receptor
